# Co-contraction of ankle muscle activity during quiet standing in individuals with incomplete spinal cord injury is associated with postural instability

**DOI:** 10.1038/s41598-021-99151-w

**Published:** 2021-10-01

**Authors:** Kai Lon Fok, Jae W. Lee, Janelle Unger, Katherine Chan, Kristin E. Musselman, Kei Masani

**Affiliations:** 1 Institute of Biomedical Engineering, University of Toronto, Toronto, ON Canada; 2 KITE-Toronto Rehabilitation Institute, University Health Network, Toronto, ON Canada; 3 Rehabilitation Sciences Institute, University of Toronto, Toronto, ON Canada; 4 Department of Physical Therapy, University of Toronto, Toronto, ON Canada

**Keywords:** Spinal cord, Spinal cord diseases

## Abstract

Previous findings indicate that co-contractions of plantarflexors and dorsiflexors during quiet standing increase the ankle mechanical joint stiffness, resulting in increased postural sway. Balance impairments in individuals with incomplete spinal cord injury (iSCI) may be due to co-contractions like in other individuals with reduced balance ability. Here we investigated the effect of co-contraction between plantar- and dorsiflexors on postural balance in individuals with iSCI (iSCI-group) and able-bodied individuals (AB-group). Thirteen able-bodied individuals and 13 individuals with iSCI were asked to perform quiet standing with their eyes open (EO) and eyes closed (EC). Kinetics and electromyograms from the tibialis anterior (TA), soleus and medial gastrocnemius were collected bilaterally. The iSCI-group exhibited more co-contractions than the AB-group (EO: 0.208% vs. 75.163%, p = 0.004; EC: 1.767% vs. 92.373%, p = 0.016). Furthermore, postural sway was larger during co-contractions than during no co-contraction in the iSCI-group (EO: 1.405 cm/s^2^ vs. 0.867 cm/s^2^, p = 0.023; EC: 1.831 cm/s^2^ vs. 1.179 cm/s^2^, p = 0.030), but no differences were found for the AB-group (EO: 0.393 cm/s^2^ vs. 0.499 cm/s^2^, p = 1.00; EC: 0.686 cm/s^2^ vs. 0.654 cm/s^2^, p = 1.00). To investigate the mechanism, we performed a computational simulation study using an inverted pendulum model and linear controllers. An increase of mechanical stiffness in the simulated iSCI-group resulted in increased postural sway (EO: 2.520 cm/s^2^ vs. 1.174 cm/s^2^, p < 0.001; EC: 4.226 cm/s^2^ vs. 1.836 cm/s^2^, p < 0.001), but not for the simulated AB-group (EO: 0.658 cm/s^2^ vs. 0.658 cm/s^2^, p = 1.00; EC: 0.943 cm/s^2^ vs. 0.926 cm/s^2^, p = 0.190). Thus, we demonstrated that co-contractions may be a compensatory strategy for individuals with iSCI to accommodate for decreased motor function, but co-contractions may result in increased ankle mechanical joint stiffness and consequently postural sway.

## Introduction

Upright standing is inherently unstable as the body centre of mass (COM) is located both in front of and high above a relatively small based of support. To control the COM during upright standing, the body acts through the ankle joint which is the critical joint for postural sway during quiet standing. Specifically, it has been shown that the ankle joint torque, which is proportional to the location of the centre of pressure (COP), highly correlates to the location of the COM^1–3^. As the body COM is in front of the ankle joint in the anterior–posterior direction during quiet standing^4^, the plantarflexor muscles are constantly active to exert an extension torque about the ankle joint, i.e., plantarflexion torque. Both the soleus (SOL) and medial gastrocnemius (MG) are the major plantarflexors that synergistically and continuously control the plantarflexion torque. Conversely, the tibialis anterior (TA) responsible for the dorsiflexion of the foot is rarely active during quiet standing in healthy young individuals^5,6^.

On the contrary in older adults, it has been reported that the TA is activated much more frequently during quiet standing, resulting in increased co-contraction of the plantar and dorsiflexors^7–9^. Co-contractions may be a strategy used by older adults to stabilize their upright posture. For the plantarflexors^10^ and dorsiflexors^11^ separately, it has been shown that increases in muscle activation increases the mechanical joint stiffness leading to increased joint stability^12^. Therefore, an increase of ankle mechanical joint stiffness may be due to the co-contractions of plantarflexors and dorsiflexors. Indeed, in the study by Carpenter et al.^13^, the TA muscle activity increases with increasing postural threat (i.e., standing on a raised platform), and was correlated with an increase of the ankle mechanical joint stiffness, as shown by the correlation between the mean TA activity and the effective stiffness of the inverted pendulum model as defined by Winter^3^, i.e. stiffness constant. However, they also reported that the increase of the ankle mechanical joint stiffness accompanied an increase of the COP’s mean power frequency, which may coincide with an increase in COP velocity although this was not shown in their study. Alternatively, Warnica et al.^14^ demonstrated that an increase in voluntary co-contractions during standing were associated with an increase of COM velocity and COP velocity. Further, Vette et al.^9^ compared the COM velocity, COM acceleration and the COP velocity during quiet standing between periods with co-contraction and periods without co-contraction in older adults and reported that these measures increased during co-contraction periods. These previous findings indicate that co-contractions of plantarflexors and dorsiflexors during quiet standing increase the ankle mechanical joint stiffness, and increase postural sway as indicated by the increases of COM velocity/acceleration and/or COP velocity. Considering that mechanical joint stiffness in general helps joint stability, it is counterintuitive that an increase of mechanical ankle joint stiffness is associated with an increase in postural sway, which usually indicates postural instability. Also, it is then unclear why older adults adopt the co-contraction strategy if this increases their postural sway.

In the current study, we further investigated the role of co-contraction in the maintenance of standing balance, by examining the ankle muscle activation strategy in individuals with incomplete spinal cord injury (iSCI). Many individuals with iSCI have impaired motor performance and varying degrees of functional limitations^15–17^. Due to partial paralysis, they tend to have muscle atrophy after iSCI^18–21^ and weakness in affected muscles^22,23^. Consequently, individuals with iSCI have reduced standing balance and increased postural sway (i.e. larger COM acceleration and COP velocity)^24,25^, and greater likelihood of falls than other neurological disease groups such as Parkinson’s disease^26^. We hypothesized that individuals with iSCI adopt the strategy of co-contraction similar to older adults to compensate for their muscle weakness at the ankle joint. Thus, the purpose of this study was to investigate ankle muscle co-contraction in individuals with iSCI, and its effect on postural stability.

Additionally, we investigated the effect of co-contraction on postural sway using a computational simulation study. Co-contractions of the plantarflexors and dorsiflexors during quiet standing may increase the ankle mechanical joint stiffness resulting in increases in the COM velocity, COM acceleration, and/or COP velocity, suggesting that an increase of ankle mechanical joint stiffness increases postural sway. Conversely, Warnica et al.^14^ reported that increasing passive joint stiffness by applying an ankle foot orthotic reduced COM velocity. These confounding results may be related to the amount of stiffness increase, as Warnica et al. used a relatively basic ankle foot orthotic with an unspecified passive stiffness increase to their participants. Therefore, more quantitative approaches are required. In the current study, the second purpose was to theoretically investigate the effect of an increase in ankle mechanical joint stiffness on postural sway in a computational simulation study. We modeled the control system of quiet standing posture using a single-link inverted pendulum, and neural/mechanical proportional-derivative controllers^2,5,27^. By increasing the mechanical stiffness in the control system, we evaluated the effect of increasing the ankle mechanical joint stiffness on their postural sway, via COM acceleration and COP velocity.

## Methods

### Experimental study

#### Participants

The data used in this study were previously collected in another work by Chan et al.^28,29^ and Unger et al.^30^. Specific inclusion criteria are presented in the Supplemental Methods. The individuals with iSCI underwent two baseline assessments, separated by 2 weeks. Here, we focus on the individuals with iSCI (iSCI-group) during their baseline 1 assessments and the age- and sex matched AB individuals (AB-group). The age-matched individuals were ± 3 years of age from the corresponding individuals with iSCI and had no medical history of neurological disorders. This reduced dataset was previously analyzed and published for another study^24^ where in total thirteen AB adults (10 females, age 57.1 ± 10.5 years), and thirteen individuals with iSCI (10 females, age 52.6 ± 13.9 years, 7.6 ± 10.1 years post-injury) were analyzed. The purpose of this previous study was to study how the MG and SOL were controlled during quiet standing in the AB- and iSCI-groups by applying the concept of cosine tuning using the ankle and knee joint torques and the concept of cosine tuning. Our current study is focused on studying the co-contraction of the ankle muscles specifically on their effect on postural control. Additionally, due to some differences in analysis, the analyzed postural sways and muscle activity within this study differs from our previous study as described later in the “Methods” section.

The recruited individuals with iSCI were American Spinal Injury Association Impairment Scale (AIS) C or D^31^, had moderate trunk control, and could stand for at least 30 s without mobility aids. A registered physical therapist performed the lower extremity manual muscle test to evaluate motor function (LE-S) of the 12 muscle groups that create movement (i.e., hip: flexors, extensors, abductors, adductors, internal and external rotators; knee: flexors and extensors; ankle: dorsiflexors, plantarflexors, invertors and evertors)^32^. Additionally, the mini Balance Evaluation Systems Test (mini-BESTest)^33^ and Community Balance and Mobility Scale (CB&M)^34^ were performed to assess balance ability. The CB&M is a test designed to evaluate balance and mobility in ambulatory individuals who have balance impairments that reduce their full engagement in community living. It has been found to have less of a ceiling effect when compared to the typical Berg Balance Scale, a better ability to capture change in these higher functioning individuals, and is a valid measure for individuals with iSCI^35^. Participants were not allowed to use a walking aid during the CB&M; as a result, one participant was unable to perform the CB&M due to this restriction and was excluded from our analyses. Lastly, gait speed was measured to provide descriptive information about the mobility status of the participants. Participants walked two lengths across a mat (14 ft) at their preferred walking speed. The details of all the clinical measurements for this data set have been previously described by Unger et al. (2020). Therefore, in this study, thirteen AB adults and twelve individuals with iSCI were analyzed, differing slightly to our previously published data^24^. The participants’ demographic and injury-related data as well as clinical scores are summarized in Table 1. There was no significant difference in the age or proportion of males between the two groups (*t* test, p = 0.366; χ^2^-test, p = 1.00). Body weight was significantly greater in individuals with iSCI (*t* test, p = 0.008). All participants gave their written informed consent to participate in the study, whose experimental procedures were approved by the Research Ethics Board of the University Health Network and University of Toronto. All experiments were performed in accordance with relevant guidelines and regulations.

**Table 1:** Summary of participant information such as age, level of impairment, time since injury, and clinical scores. *LE* lower extremity, *mini-BESTest* mini-Balance Evaluation Systems Test, *CB&M* Community Balance and Mobility Scale, *C* cervical, *T* thoracic, *L* lumbar, *4WW* 4-wheeled walker. ^d^Denotes participant excluded from data analyses, due to inability to complete CB&M assessment. *LE strength measured with manual muscle testing of 12 muscles per LE. Maximum score per muscle is 5, resulting in a total score of 120 for 2 LE. ^†^Denotes retrospective falls in the previous 3 months.

	*PBT05*	*PBT08*	*PBT10*	*PBT13*	*PBT14*	*PBT16*	*PBT17*^d^	*PBT18*	*PBT20*	*PBT22*	*PBT23*	*PBT24*	*PBT25*	Mean (SD)
Sex	F	M	F	F	F	F	F	F	F	M	F	M	F	
Age (years)	32	60	43	57	59	55	38	54	56	88	38	51	53	52.6 (13.9)
Weight (kg)	49.9	109.2	47.3	102.0	62.3	47.5	55.6	83.3	73.7	77.2	79.9	81.9	68.9	72.2 (19.7)
Level of Injury	C4	C5	T6	C2	C1	C5	T4	C4	L5	C6	T11	C3	C4	
Time since injury (years)	3.5	3.2	3.9	2.9	1.1	9.1	1.3	13	1.2	5.3	6.8	7.9	39	7.6 (10.1)
LE strength (/120)*	87.5	115	104.5	89	75	90	75	78.5	70	81.5	101.5	97	89.5	88.8 (13.1)
Left plantarflexor LE score (/10)	7.75	9.5	9.25	7.25	7	9.25	7	6.5	6.25	7	8.75	10	7	7.9 (1.3)
Right plantarflexor LE score (/10)	7.75	10	9.5	7.75	7	9.25	7	7.75	7	5.5	9.5	7.75	8	8.0 (1.3)
Usual walking aid	None	None	None	Cane	4WW	Cane	4WW	Cane	None	None	None	Poles/4WW	Poles	
Mini-BESTest Score (/28)	25	25	24	21	4	25	5	13	17	12	22	15	15	17.2 (7.3)
Gait speed without aid (m/s)	1.29	1.28	1.10	0.72	0.43	0.88	0.75	0.91	0.94	0.83	1.03	1.29	0.95	0.954 (0.251)
CB&M (/96)	89	70	78	29	3	26	N/A	27	33	20	63	52	33	40.2 (27.9)
Fall history^†^	1	0	1	0	0	1	1	0	0	0	1	0	1	

	*AB1*	*AB2*	*AB3*	*AB4*	*AB5*	*AB6*	*AB7*	*AB8*	*AB9*	*AB10*	*AB11*	*AB12*	*AB13*	Mean (SD)
Sex	F	F	F	M	F	F	M	F	F	F	F	M	F	
Age (yrs)	54	57	47	67	59	53	57	84	62	56	55	51	40	57.1 (10.5)
Weight (kg)	61.9	42.3	55.5	57.4	49.6	45	75.4	43.9	55.7	44.7	45.3	69.9	53.6	53.9 (10.4)

#### Procedure

Participants performed two quiet standing trials, with eyes open (EO) and eyes closed (EC). For each condition, participants stood quietly in shoes with their arms across their chest for 150 s. By placing arms across their chest, we reduce the degrees of freedom during standing and more closely resemble the inverted pendulum model as described by Winter^3^ used in our simulations of quiet standing (described later under Simulation study). A rest was taken if needed between conditions to minimize the effect of fatigue. In the EO condition, participants were instructed to focus on a visual target placed at eye level and 2 m in front of them for the duration of the trial. All participants wore a safety harness that was connected to the ceiling to prevent participant falls for all trials. The harness was set up such that it did not support the weight of participants during standing tasks. A dual force plate (AccuSway ACS-DUAL, Advanced Mechanical Technology, Watertown, MA) was used to measure the ground reaction force during quiet standing.

Surface EMGs were recorded from SOL, MG, TA, and other muscles bilaterally, though we only focus on SOL, MG and TA in this study. For the AB-group, we analyzed only left muscles under an assumption of symmetry. For the iSCI-group, we analyzed both sides, and we identified the strong leg for the group summary, based on the total SOL and MG LE-S. That is, the SOL score was taken as the measured plantarflexion LE-S, and the MG score was taken as the average of plantarflexion and knee flexion LE-S. The combined SOL and MG scores were used to determine the strong leg. When the sum of the SOL and MG scores for both legs were equal, the left leg was chosen as the strongest (4 participants out of 13). These scores are summarized in Table 1. The EMGs were sampled, and the raw signal was amplified and hardware band-pass filtered between 20 and 500 Hz (Bagnoli 8 EMG System, Delsys, Boston, MA). The force plate data and EMG data were sampled at 2 kHz and stored on a personal computer for subsequent analysis. Prior to the standing trials, resting EMG levels were recorded in a seated posture for 30 s. Further, two maximum voluntary contractions (MVC) trials were collected for each lower limb muscle, details of the posture, contraction durations and absolute values for the MVC trials are described in the Supplementary Methods and Supplementary Tables S1 and S2.

#### Data processing

All data processing was performed using computational software (MATLAB 2018a, MathWorks, USA). We focused on the anterior–posterior direction given that body movement during quiet standing is primarily in this direction and that plantarflexors primarily contribute to body motion in this direction. For each 150-s trial, the first and last 15 s of the time series data were trimmed to ensure analysis of steady state standing, which yielded the analyzed period of 120 s. After trimming, the remaining 120 s were separated into two 60 s windows to minimize the effect of subtle body shifts over time. The subsequent analyses were applied for each 60-s window, and the average of the two parameters was used for each participant’s parameter.

The ground reaction force components obtained from the force plate were 4th-order Butterworth low-pass, zero-phase-lag filter with a cut-off frequency 4 Hz since the study focuses on muscle activity during low frequency body movements^2,36^. To quantify the postural sway during quiet standing, the COP displacement was first calculated using the ground reaction force. Then the centre-of-pressure velocity (COPv) was calculated as a measure of postural sway. Additionally, the centre-of-mass acceleration (COMa) was calculated using the horizontal ground reaction force component according to: ${COM}_{ACC}={f}_{AP}/m$ where ${f}_{AP}$ is the horizontal force in the anterior–posterior direction, and *m* is the participant’s body mass (*M*) excluding the weight of their feet (*m* = 0.971 M)^37^. The calculation of the COMa required an additional high pass filter at 0.15 Hz (essentially becoming bandpass filtered) to exclude the trend of the horizontal ground reaction force component^38,39^. The standard deviation (SD) of COPv and COMa were used to quantify the postural sway for each participant.

The EMGs of the SOL, MG and TA were rectified and smoothed using a 4th-order Butterworth low-pass, zero-phase-lag filter with a cut-off frequency of 1 Hz^9^ to estimate muscle activity levels. MVC recordings for the SOL, MG and TA were filtered using the same parameters as above, and the MVC was chosen as the maximum value during the 3–4 s sustained contraction period. The periods of TA activity (TA*on*) and inactivity (TA*off*) were identified from the smoothed time series data in which the TA EMG activity exceeded 2.5% of the corresponding TA MVC. In our previous study, the resting threshold plus 3 SD had been used to identify periods of activity and inactivity^9^; however, in our resting EMG data, we found that individuals with iSCI had difficulty in fully relaxing their muscles impacting their resting threshold value (e.g., in some cases the resting EMG was larger than EMG during standing). To determine an appropriate threshold for identifying TA*on* periods, we examined four percent MVC thresholds of 1.5, 2, 2.5 and 3% as shown in Supplemental Fig. S1 online. At thresholds of 2% and below, the AB-group’s TA is nearly always on. These periods are unexpected as previous studies have reported infrequent TA activity in young able-bodied individuals^5,6^. Therefore, a threshold of 2% was not selected to determined periods of TA*on* due to the difference in previously reported periods of TA activity. When a threshold of 2.5% was used, the trends of TA activity for the AB-group matched previous reports of comparable TA activity. Furthermore, 2.5% was found to be around the median of the AB-group’s TA EMG activity during quiet standing. Thus, 2.5% was chosen as the threshold amount.

#### Data analyses

The COP and COM data are non-stationary over shorter periods of time due to their fractal properties^40^, resulting in increased variability with increased data length. Consequently, results associated with COP and COM variability are highly biased and unreliable when comparing periods of significantly different lengths. Alternatively, the COPv and COMa are rather stationary signals in both the short- and long-term, and the data length of these measures will not significantly impact the quantified variability. Further, both the COPv and COMa have been used to evaluate standing balance ability in children, young able-bodied adults and the elderly while also classifying fallers from non-fallers^41–44^. Thus, in this study, the SD of the COPv and COMa were calculated as measures of postural sway. The COPv and COMa during the entire standing trial, and during TA*on* and *TAoff* were calculated for each group and condition. The periods of TA*on* and TA*off* were chosen to analyze as periods of co-contraction and no co-contraction respectively because despite TA activity the antagonists (i.e., MG and SOL) did not become inactive (Supplemental Fig. S2). In fact, during quiet standing both plantarflexors are active for ~ 100% of the duration, thus during periods of TA*on* both the plantar- and dorsiflexors should be co-contracted. Conversely, during TA*off* periods only the plantarflexors are active. The magnitude of muscle activity level was represented as the percent MVC (%MVC) by identifying the mean EMG level during periods of quiet standing above 2.5% MVC of the respective muscle as a percentage of the muscle’s MVC. The variability of each muscle activity level was quantified as the coefficient of variation (CV) for the entire quiet standing trial. The percent duration of activity of each muscle (%On) during quiet standing was calculated as the percentage of the trial the EMG activity level was greater than 2.5% of its MVC. Similarly, the percent duration of co-contraction (%CC) of the SOL/TA was calculated as the percentage of the trial where both the SOL and TA were greater than 2.5% of their respective MVCs.

The normality of each measure was tested using the Shapiro–Wilk test. We found that the COPv and COMa for the entire quiet standing trial were normally distributed. Two-factor mixed analysis of variance (ANOVA) tests (*vision* [EO/EC] × *group* [AB/iSCI]) were used to examine the effect of vision and the groups on the COPv and COMa. The remaining measures (%MVC, CV, %On, %CC, COPv and COMa during TA*on and* TA*off*) were non-normally distributed. Thus, for the %MVC, CV, %On, and %MVC on/off ratio, a Wilcoxon’s rank-sum test (non-paired) was used to compared significant differences between groups, for each condition respectively. The COPv and COMa during TA*on* and TA*off* were compared for each group and condition separately using a Wilcoxon’s signed-rank test (paired). Bonferroni corrections were used for all multiple comparisons. A statistical software (SPSS Statistics ver. 25, IBM Corp., USA) was used for all statistical tests. *p* < 0.05 served as the significance level.

### Simulation study

#### Model

**Figure 1:**
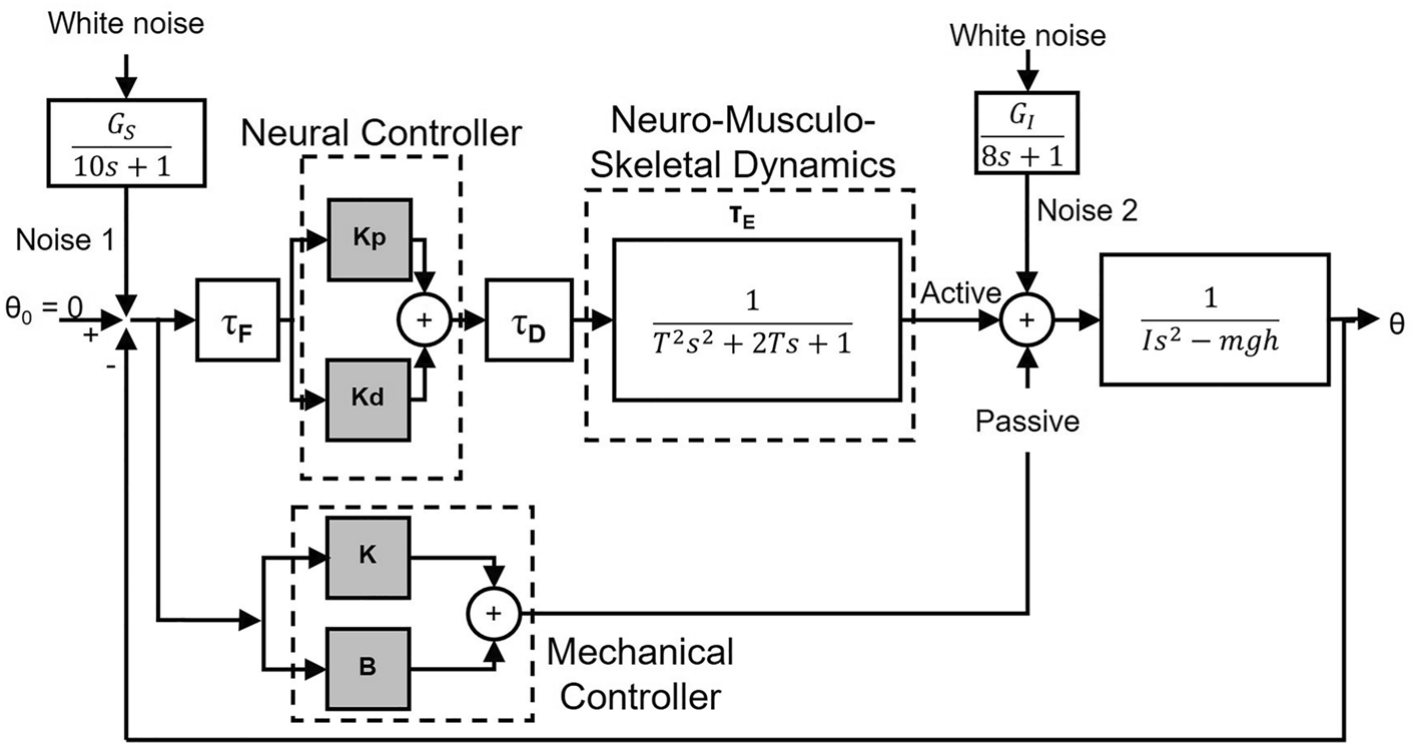
Computational model used for simulating the control system of quiet standing. The model consisted of a neural controller using a proportional-derivation (PD) controller with gains *Kp*, and *Kd,* and a mechanical controller using a PD controller with gains *K* and *B*. A feedback time delay (τ_F_ = 40 ms) and motor command time delay (τ_D_ = 40 ms) were placed within the neural controller^47,48^. After this delay, a critically damped, 2nd order model of the neuromuscular system was inserted to account for the ankle torque generation process producing the neural torque component. An inverted pendulum was used as the model of the standing body. Two first order filtered white noise inputs (G_S_ and G_I_) corresponding to the sensory and neuro-mechanical noise were injected into the system to drive the simulation.

The computational simulation study was performed using MATLAB (ver. 9.5, MathWorks, United States) and Simulink (ver. 9.2, MathWorks, United States). A computational model representing the control system of quiet standing was developed (Fig. 1) based on previous work on healthy young able-bodied participants^2,5,27,45^. This model was chosen to focus on the effect of an increase of ankle mechanical joint stiffness as we hypothesized this occurs during the co-contraction of plantar- and dorsiflexors. The model consisted of a neural controller consisting of a proportional gain, Kp and derivative gain, Kd which provide the active torque components. The mechanical controller provided the rotational stiffness K and rotational viscosity B, i.e., passive torque components. Based on Loram et al.^46^, B was set to 5 Nm s/rad. A feedback time delay (τ_F_ = 40 ms)^47^ and motor command time delay (τ_D_ = 40 ms)^48^ were placed within the neural controller. After this delay, a critically damped, 2nd order model of the neuromuscular system was inserted to account for the ankle torque generation process producing the active neural torque component^5^. Three different muscle twitch contraction periods were used: 0.121, 0.152, and 0.192 s^5^. An inverted pendulum was used as the model of the standing body. The average mass of the AB- and iSCI-group was 63.1 kg. Given the large SD for each group, an inverted pendulum mass of 59.2 kg was chosen based on a previously published inverted pendulum^45^. The other parameters for the inverted pendulum were also taken from the previous study as: h = 0.846 m, m = 59.2 kg, I = 55.2 kg m^2^^45^, where h is the height of the COM, m is the mass of the pendulum, and I is the moment of inertia. These parameters (i.e., m, h, and I) were kept consistent across all simulations. Two noise inputs corresponding to the sensory and neuro-mechanical noise were injected into the system (Fig. 1) to drive the simulation. The noise injected at two different locations in the feedback loop, represented white noise filtered with 10 and 8 s for the noise 1 (sensory) and noise 2 (internal) respectively. These time constants were chosen because they produced waveforms with sway characteristics like those seen in experiments.

#### Simulation and analysis

To determine the cause of differences in postural sway in the AB- and iSCI-groups, we ran simulations that represented both groups and visual conditions. This was achieved through noise driven simulations run using random values generated by the *rand* function in MATLAB along with four different noise gain combinations that were identified to correspond to a group and standing condition (e.g. AB-group EO). The gains for sensory and internal noises were identified by running the simulations with various gain combinations and matching the COPv and COMa values to the experimental data values of the AB- and iSCI-groups for each visual condition. The following represents the identified noise gain combinations: for the AB-group in EO condition, sensory noise gain (G_S_) = 1.20, internal noise gain (G_I_) = 4.80; for the AB-group in EC condition, G_S_ = 1.70, G_I_ = 6.80; for the iSCI-group in EO condition, G_S_ = 2.25, G_I_ = 9.00; for the iSCI-group in EC condition, G_S_ = 3.60, G_I_ = 14.4.

Further, as co-contractions are thought to increase the mechanical joint stiffness, we investigated the effect of co-contractions on postural sway by evaluating postural sway at one stiffness K, followed by an increased stiffness using the same parameters. To achieve this, we systematically changed Kp, Kd and K in the ranges of 0 ≤ *Kp* ≤ 500 Nm/rad, 100 ≤ *Kd* ≤ 300 Nm s/rad, and 100 ≤ *K* ≤ 1000 Nm/rad with a step size of 50 for each controller variable. From here onwards, simulations run with noise gains matching the AB-group and iSCI-group are referred to as Sim-AB and Sim-iSCI, respectively. The simulations with an increased K parameter are referred to as Sim-AB-CC and Sim-iSCI-CC for noise matched AB- and iSCI-groups, respectively. The ratio of the different increases in K for the AB-group and iSCI-group was based on the experimental data (Supplementary Fig. S3). However, as we did not measure or analyze the exact ankle stiffness during quiet standing, the specific increase values, 1% and 70% were chosen arbitrarily to represent a minimal stiffness increase and large stiffness increases for the two groups. For both Sim-AB/Sim-iSCI and Sim-AB-CC/Sim-iSCI-CC simulations, the stability of the system was investigated using the Nyquist stability criterion with gain and phase margins of 2 dB and 5° respectively. Only systems that met these stability criteria for both the Sim-AB/Sim-iSCI and Sim-AB-CC/Sim-iSCI-CC simulation parameter combinations were used, resulting in 137 gain combinations for the Sim-AB/Sim-AB-CC simulations and in 55 gain combinations for the Sim-iSCI/Sim-iSCI-CC simulations. The identified gains that stabilized the system according to the gain and phase margin criteria had the following ranges: 0 ≤ *Kp* ≤ 350 Nm/rad, 100 ≤ *Kd* ≤ 250 Nm s/rad, 300 ≤ *K* ≤ 1000 Nm/rad, and muscle twitch contraction periods of 0.121 and 0.152 s for the Sim-AB/Sim-AB-CC simulations, and 0 ≤ *Kp* ≤ 200 Nm/rad, 100 ≤ *Kd* ≤ 200 Nm s/rad, 450 ≤ *K* ≤ 1000 Nm/rad, and muscle twitch contraction periods of 0.121 and 0.152 s for the Sim-iSCI/Sim-iSCI-CC simulations.

After, the identified stable control systems were used to simulate postural sway during quiet standing for 70 s. The first 10 s of the data was trimmed to ensure observation of steady-state responses. The COPv and COMa were calculated based on the motion of the inverted pendulum. A low-pass, zero-phase-lag filter with a cut-off frequency of 4 Hz using a 4th-order Butterworth was applied to both the COPv and COMa. Subsequently, the fluctuation amount of the COPv and COMa were quantified using SD for the entire 60 s as measures of postural sway.

A Wilcoxon signed-rank test was used to test for significant differences between Sim-AB/Sim-iSCI and Sim-AB-CC/Sim-iSCI-CC simulations for the COPv and COMa fluctuations for each set of noise parameters. *p* < 0.05 served as the level of statistical significance.

## Results

### Experimental study

**Figure 2:**
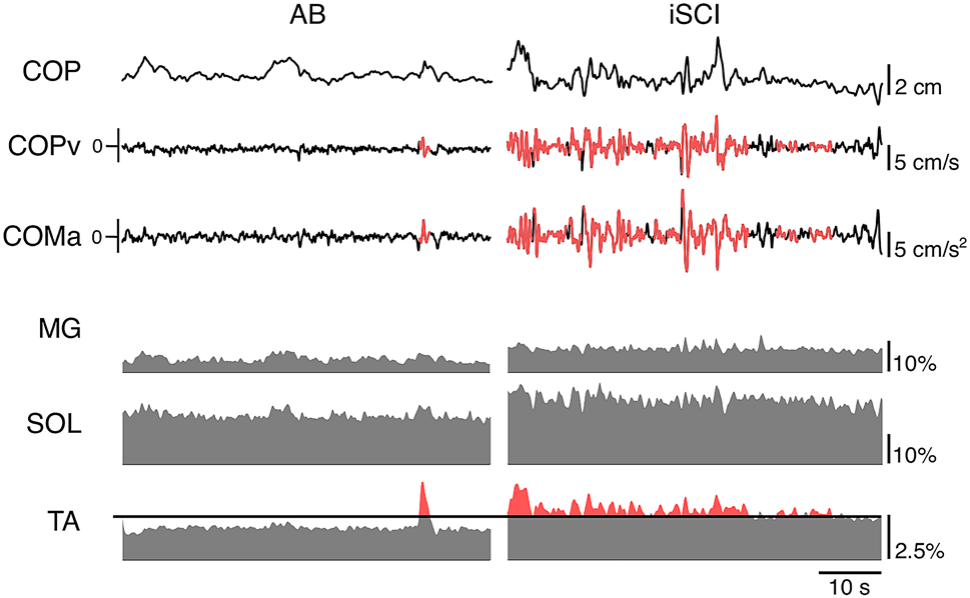
Representative traces of 60 s of the measured variables during quiet standing for both AB- and iSCI-groups. Specifically, the COP, COPv, and COMa are plotted for the AB-group, and iSCI-group in the first three rows. The last three rows are the EMG activity of the MG, SOL and TA. The horizontal black line across the TA row represents the activation threshold of 2.5%MVC. Periods where the TA EMG activity was greater than this threshold are shown in red, and the corresponding COPv and COMa periods are shown in red as well.

Typical examples of the time series data for the force plate-based and EMG measures are shown for the AB- and iSCI-groups in Fig. 2. In general, the COPv and COMa appear to be larger for the iSCI-group than the AB-group. Further, Fig. 2 illustrates the greater level of TA activity of the iSCI-group when compared to the AB-group.

#### Postural control

**Figure 3:**
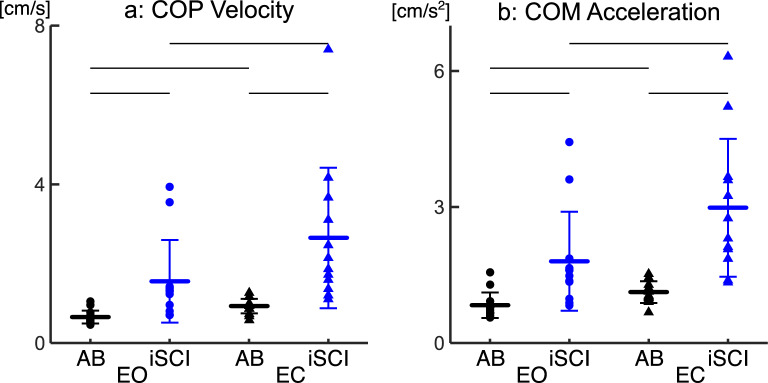
Comparison of the postural sway (standard deviation of the COPv and COMa) during quiet standing between the AB- and iSCI-group. (**a**) Distribution of the COPv fluctuations in the AP direction for EO and EC conditions. (**b**) Distribution of the COMa in the AP direction for EO and EC conditions. Thick blue and black horizontal lines represent the group mean. Thin vertical lines represent the standard deviation of about the mean. Thin black horizontal bar indicates a p-value < 0.05 between the two groups under each condition (AB-group: N = 13—black, iSCI-group: N = 12—blue). Circles are EO condition, EC conditions are triangles.

Figure 3 shows the group results for the COPv and COMa for the entire standing task (Fig. 3). For COPv, a two-way mixed ANOVA revealed the significant main effects of group (F(1,23) = 11.433, p = 0.003, η^2^ = 0.332) and condition (F(1,23) = 25.745, p < 0.001, η^2^ = 0.528). A significant interaction (F(1,23) = 9.204, p = 0.006, η^2^ = 0.286) was also found. The post-hoc test revealed that the COPv was significantly larger in the iSCI-group than in the AB-group for both conditions (EO: p = 0.024, EC: p = 0.008). For both groups, the COPv was significantly larger in EC condition than EO condition (iSCI-group: p = 0.08, AB-group: p < 0.001) (Fig. 3a).

For COMa, a two-way mixed ANOVA revealed a significant main effect of group (F(1,23) = 15.577, p = 0.001, η^2^ = 0.404) and condition (F(1,23) = 43.477, p < 0.001, η^2^ = 0.654). A significant interaction (F(1,23) = 15.965, p = 0.001, η^2^ = 0.410) was also found. The COMa was significantly larger in the iSCI-group than the AB-group for both conditions (EO: p = 0.020, EC: p < 0.001). For both groups, the COMa was significantly larger in the EC condition than in the EO condition (iSCI-group: p < 0.001, AB-group: p = 0.012) (Fig. 3b).

#### Muscle activity

**Figure 4:**
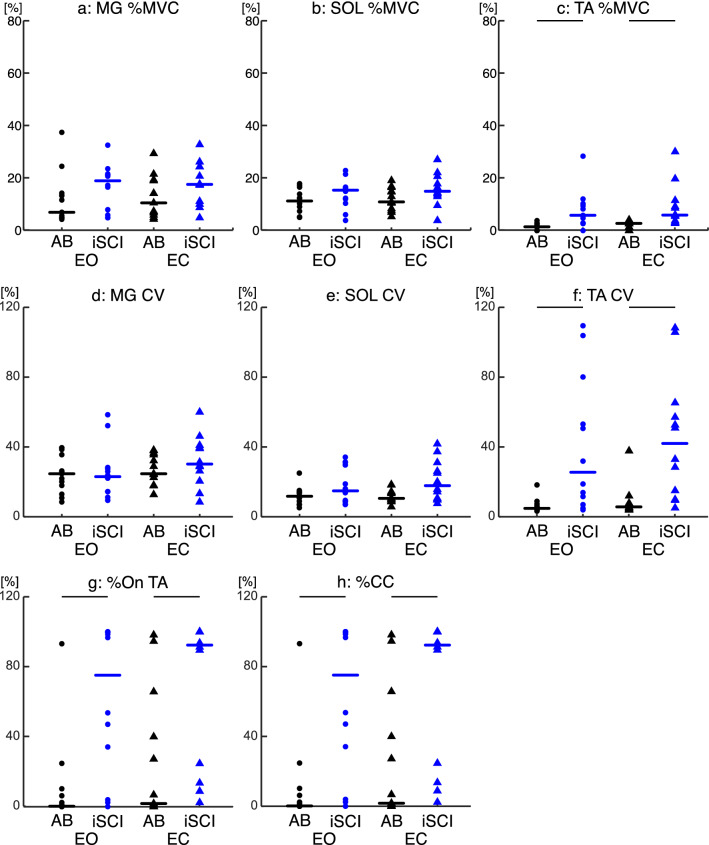
Comparison of measures of EMG (%MVC, CV, %On and %CC) between the AB- and iSCI-group. (**a**) Distribution of the mean MG EMG activity as a percentage of the MVC. (**b**) Distribution of the mean SOL EMG activity as a percentage of the MVC. (**c**) Distribution of the mean TA EMG activity as a percentage of the MVC. (**d**) Distribution of the mean MG CV. (**e**) Distribution of the mean SOL CV. (**f**) Distribution of the mean TA CV. (**g**) Distribution of the mean amount of time TA was active as a percentage. (**h**) Distribution of the mean amount of time the SOL and TA were active as a percentage. Group data presented for the AB- (black) and the iSCI-group (blue) in both EO and EC conditions. Individual participant data are plotted as circles for the EO condition and triangles for the EC condition. N = 13 for AB-group, and N = 12 for iSCI-groups. Thick black, blue, and red horizontal lines represent the group median. Thin black horizontal bar indicates a p-value < 0.05.

Figure 4 shows the mean muscle activation results for each muscle when above 2.5%MVC of their respective MVC. Overall, the iSCI-group demonstrated greater muscle activation (%MVC), variability (CV) and co-contraction (%CC) than the AB-group. For each of EO and EC conditions, the %MVC was not different between the AB- and the iSCI-groups for the MG and SOL (MG: EO—p = 0.173, EC—p = 0.242; SOL: EO—p = 0.442, EC—p = 0.242) (Fig. 4a,b). For the TA %MVC, the %MVC was significantly different between the AB- and the iSCI-group for each of EO and EC conditions (EO, p = 0.001; EC, p < 0.001) (Fig. 4c).

The CV was not significantly different between the AB- and the iSCI-groups for each of EO and EC conditions for the MG and SOL (MG: EO—p = 1.00, EC—p = 0.530, SOL: EO—p = 0.442, EC—p = 0.094). For the TA CV the iSCI-group than the AB-group was significantly larger for both EO and EC (EO—p = 0.004, EC—p = 0.001) (Fig. 4d–f).

Figure 4g,h shows the period of the TA’s activity and SOL/TA co-contractions. The TA %On was significantly larger in the iSCI-group (EO—p = 0.004, EC—p = 0.016) than in the AB-group for both conditions (Fig. 4g). The median value and range of TA*on* durations for both groups is shown in Supplementary Table S3 online. Also, the %CC was significantly larger in the iSCI-group than in the AB-group in both EO and EC conditions (EO—p = 0.004, EC—p = 0.016) (Fig. 4h). Results of the group median results for the MG and SOL %On and MG/TA co-contraction are presented in Supplementary Figure S2 online.

#### Effect of TA activation on postural sway

**Figure 5:**
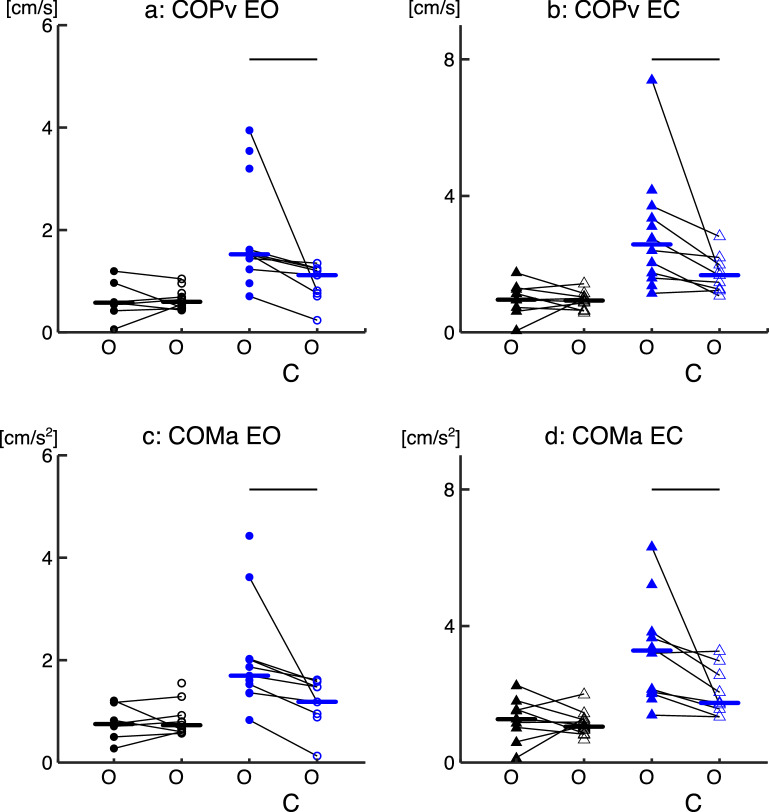
Comparison of the postural sway (standard deviation of the COPv and COMa) during TA*on* and TA*off* between the AB- and iSCI-group. (**a**) Distribution of the fluctuation of the COPv in the EO. (**b**) Distribution of the fluctuation of the COPv in the EC. (**c**) Distribution of the fluctuation of the COMa in the EO. (**d**) Distribution of the COMa in the EC condition. Whole body COPv and COMa group data presented for the AB-group (black) and iSCI-group (blue) in both EO (circles) and EC (triangles) conditions for periods of co-contraction (On—filled shape) and no-co-contraction (Off—open shape). Horizontal lines represent the group median. Each point represents an individual participant’s data, thin black lines between data points connect the same participant and thin horizontal lines above the plots represent significant differences (p < 0.05). Thick horizontal lines within data points represent group medians.

Figure 5 shows the results of COPv and COMa during TA*on* and TA*off*. During TA*on*, the AB-group demonstrated no changes in postural sway; however, the iSCI-group’s postural sway increased significantly. Specifically, the COPv was not significantly different between TA*on* and TA*off* in the AB-group in EO (p = 1.00) and EC (p = 1.00) conditions. In the iSCI-group, during TA*on* the COPv was significantly larger than during TA*off* in EO (p = 0.023) and EC (p = 0.022) conditions (Fig. 5a,b). The COMa was not significantly different between TA*on* and TA*off* periods for the AB-group in EO (p = 1.00) and EC (p = 1.00) conditions. In the iSCI-group, during TA*on* periods, the COMa was significantly larger than during TA*off* periods in EO (p = 0.023) and EC (p = 0.030) conditions (Fig. 5c,d). A similar trend can be observed when calculating the COPv and COMa for the corresponding single leg as shown in Supplementary Fig. S4 online.

### Simulation study

**Figure 6:**
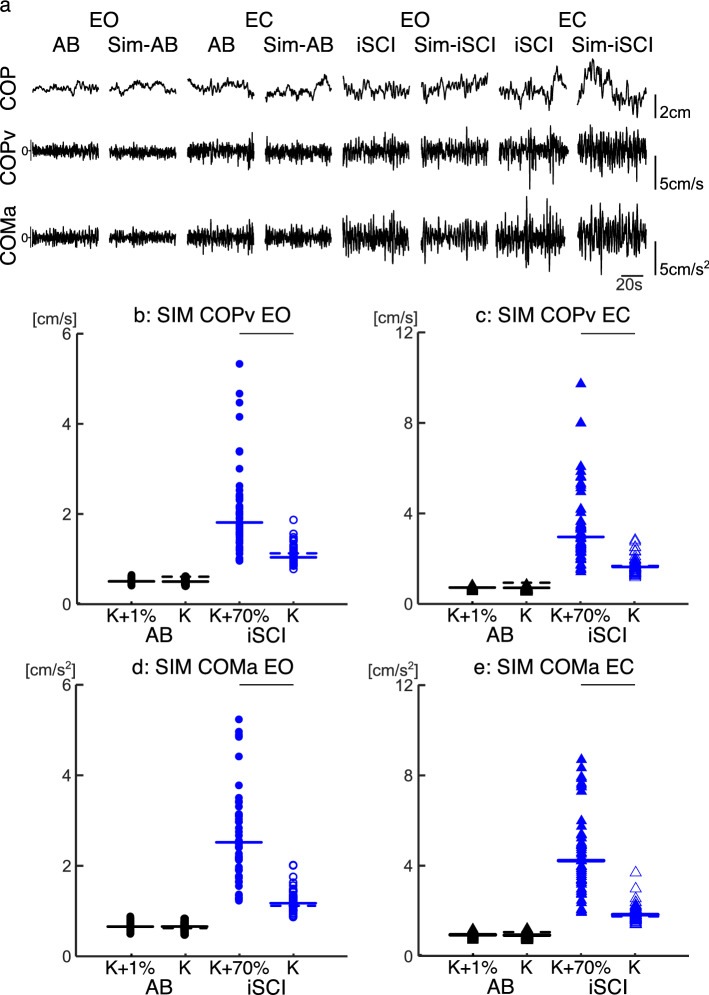
Example of the simulated postural sway (standard deviation of the COPv and COMa), and the comparison of an experimentally matched postural sway to postural sway due to an increased mechanical stiffness. (**a**) Representative 60 s traces for the experimental (AB/iSCI) and simulated (Sim-AB/Sim-iSCI) COP, COPv and COMa in both EO and EC conditions. (**b**) Distribution of the fluctuation of the COPv in the EO condition. (**b**) Distribution of the fluctuation of the COPv in the EC condition. (**c**) Distribution of the fluctuation of the COMa in the EO condition. (**d**) Distribution of the COMa in the EC condition. Simulation data presented for the Sim-AB (black, N = 137) and Sim-iSCI (blue, N = 55) groups in both EO (circles) and EC (triangles) conditions for periods of co-contraction (K + %—filled shapes) and no-co-contraction (K—empty shapes). Thick horizontal lines represent the group median, horizontal dashed lines represent the target experimental postural sway data used to determine the gains sensory and internal noise for the simulations. Individual data are plotted. Thin horizontal lines above the plots represent significant differences (p < 0.05).

In total, 1072 simulations were executed. Figure 6a shows representative time series of the matched COP, COPv and COMa to the experimental data for both groups, and EO and EC conditions. Figure 6b–e also shows the COPv and COMa of the matched experimental noise gains and the resultant mechanical stiffness increase. Overall, for the Sim-AB noise gains, no significant differences were found between Sim-AB and Sim-AB-CC simulations whereas for the Sim-iSCI noise gains the postural sway significantly increased in the Sim-iSCI-CC simulations. For the COPv, there were no significant differences between the Sim-AB and Sim-AB-CC simulations in the EO (p = 1.00) or EC (p = 0.246) conditions. The Sim-iSCI-CC simulation COPv was significantly larger than the Sim-iSCI COPv in both EO (p < 0.001) and EC (p < 0.001) conditions (Fig. 6b,c). For the COMa, there were no significant differences between the Sim-AB and Sim-AB-CC simulations in the EO (p = 1.00) or EC (p = 0.190) conditions. The Sim-iSCI-CC simulation COMa was significantly larger than the Sim-iSCI COMa in both EO (p < 0.001) and EC (p < 0.001) conditions (Fig. 6d,e).

## Discussion

### Increases in TA muscle activity in individuals with iSCI

We demonstrated that individuals with iSCI show larger COP velocity and COM acceleration during quiet standing (Fig. 3) in agreement with previous studies^24,25^. Further, our results demonstrated that SOL and MG %MVC as well as CV were similar between the AB- and iSCI-groups (Fig. 4a,b,d,e), while TA %MVC and CV were larger in the iSCI-group (Fig. 4c,f). The results of the COP velocity, COM acceleration, %MVC and MG/SOL CV are similar to our previous study using the same data set. However, these results are unique given the smaller sample size of the iSCI-group in the current study and the selection of EMGs from the strong leg in the current study rather than the left leg in our previous study^24^. Also, the period of co-contraction, %CC, was larger in the iSCI-group compared to the AB-group (Fig. 4h). These results indicate that they used co-contractions more compared to the AB-group. This supports our first hypothesis that individuals with iSCI adopt the strategy of co-contraction probably as a compensatory strategy similar to older adults for their muscle weakness at the ankle joint.

Due to the tendency for muscle atrophy after iSCI^18–21^ there is a decrease of strength in plantarflexors^49^. For individuals with iSCI, they experience muscle weakness in controlling the foot while maintaining dexterity. Specifically, individuals with iSCI exhibit decreased maximal ankle movement velocity which strongly correlated with their motor scores, but they had no difficulty in accurately moving their foot rhythmically to an auditory cue^50–53^. These findings indicate that the individuals with iSCI may maintain good control of the available motor units, but the number of muscle fibres are limited. Therefore, we expected that individuals with iSCI would show a relatively higher effort and activity (i.e., %MVC and CV) in the plantarflexors to compensate for the limited number of muscle fibres. However, this was not the case; the effort and activity of the plantarflexors in the individuals with iSCI were not significantly different from the ones in AB participants while there was a tendency of increase (Fig. 4). Also, the strength of plantarflexor (i.e., plantarflexor LE-S) did not correlate with the SD of COPv or COMa (p = 0.069 to 0.306, not shown in the results). These results suggest that the weakness of plantarflexors may not be the primary factor of large postural sway in individuals with iSCI. One reason why the weakness of the plantarflexors did not primarily determine the large amount of postural sway may be due to the strategy involving co-contractions that individuals with iSCI adopt, where the ankle joint control is not primarily determined by plantarflexor activity. This may suggest that an adoption of a co-contraction strategy may mask weaknesses in plantarflexor muscles. As discussed in the next sub-section, the amount of co-contraction actually determines the amount of postural sway.

The increased co-contraction in the iSCI-group may be due to a switch of reciprocal inhibition to facilitation. Both recurrent and presynaptic inhibition operate incorrectly after iSCI^54–56^ which influences reciprocal inhibition. Indeed, previous studies have demonstrated that after iSCI, reciprocal inhibition is replaced with facilitation^57–61^ which may lead to the increase in co-contractions between the plantarflexors and TA. While this was not tested in the current study, it would be interesting and informative to perform further studies testing the correlation between the level of reciprocal inhibition between the TA and SOL and the amount of co-contraction during quiet standing. Overall, the increase of co-contractions between the plantarflexors and the TA after iSCI may be a compensation mechanism for decreased leg strength or compromised neural circuits.

### Effects of co-contraction on postural sway

A previous study by Vette et al. (2017) demonstrated an increase in postural sway, via COM velocity/acceleration and COP velocity, during periods of co-contraction in older adults, but no such increases were found for the young group. Here, we demonstrated a similar finding that during periods of co-contraction the COPv and COMa were significantly larger than periods of no co-contraction for the iSCI-group, but not for the AB-group (Fig. 5). Our results suggest that ankle muscle co-contractions are associated with increased postural sway in individuals with iSCI, while the co-contraction in the AB-group did not increase the postural sway, agreeing with our previous study in the young and older adults.

These findings partially agree with the findings of Warnica et al.^14^ who demonstrated increased voluntary co-contractions (~ 30–40%MVC) of muscles around their ankles during standing corresponded with increases in COM and COP velocities. However, this level of voluntary co-contraction is typically not seen during quiet standing in young or older adults^8^. In our AB-group, the strength of co-contraction observed was lower than 30–40%MVC (i.e., muscle activation of plantarflexors and dorsiflexor activity were lower than this level) (Fig. 4a–c) which more closely resembles previously reported ranges^8^. However, in the iSCI-group, the plantarflexors and TA were closer to the 30–40%MVC range (Fig. 4a–c). Consequently, as the amount of muscle activation relates to the mechanical joint stiffness, the ankle joint stiffness in iSCI-group must be larger than that of AB-group. Thus, the strength of co-contraction was stronger in iSCI-group which closely resembled the strength of co-contraction used in Warnica et al. (2014), while the strength of co-contraction in the AB-group was weaker than the strength in Warnica et al. (2014). For example, Sinkjaer et al.^11^ found that the total ankle mechanical joint stiffness increased by about 400% when the strength of the TA activity was between 30–80% MVC with no active plantarflexor activity. Further, it has been reported that individuals with iSCI exhibit greater ankle mechanical joint stiffness than AB individuals^62^. Therefore, the stronger co-contraction in individuals with iSCI yielded a larger mechanical joint stiffness at a similar level to Warnica’s condition, resulting in the larger postural sway, while this is not the case as the strength of co-contraction was weak in the AB-group.

One may think that postural sway may be less when the joint stiffness is larger, because in general co-contractions are supposed to stabilize the body movement instead of worsening it However, Warnica et al. (2014) reported that as muscle activation levels of the plantarflexor and dorsiflexors increased (i.e., strength of co-contraction) the COM and COP velocity increased, but an increase of passive stiffness via a foot orthotic reduced COM velocity. Thus, the level of stiffness may be one of the critical factors that positively or negatively determines the amount of postural sway. To test this, we conducted a computational simulation study to quantitatively show this assumption. First, we identified the appropriate internal noise parameters for each standing condition and group, considering the possible decrease in proprioceptive ability in the iSCI-group (Fig. 3)^25^. Then, a different mechanical stiffness increase was applied to AB- and iSCI-group simulations to accommodate for the different strengths of co-contraction (Supplementary Fig. S3). That is, a small mechanical stiffness increase (1%) was chosen for the Sim-AB-CC to represent a small increase in the TA %MVC during TA*on* and TA*off* periods, while a larger 70% mechanical stiffness increase was used for the Sim-iSCI-CC. We found that Sim-iSCI-CC had significantly larger COPv and COMa than the Sim-iSCI, but no such differences were observed for Sim-AB-CC and Sim-AB simulations (Fig. 6b–e). These results correspond to the results observed in our experimental data for the AB- and iSCI-groups. Thus, the increased postural sway seen during co-contractions in the iSCI-group may have been a result of significantly increased ankle mechanical joint stiffness due to a large increase in the amount of TA activity. On the contrary, no such increases in postural sway during co-contraction were seen in the AB-group due to the small increase of mechanical stiffness during periods of co-contraction. These results suggest that after iSCI the coordination of muscle activation may be affected resulting in greater postural sway. Indeed, our previous work has indicated an altered control strategy of the MG when considering the ankle and knee joint torques. Also, as mentioned previously, previous studies have demonstrated that after iSCI, reciprocal inhibition is replaced with facilitation^57–61^. The results of this study suggest then that the altered control strategies of the MG and neural circuits may need to be a greater priority for rehabilitation practices. Therapies capable of improving/restoring the appropriate spinal neural circuit connections may result in a greater improvement in standing balance for individuals with iSCI than current options. In summary, the results of this study may help with the development of rehabilitation techniques and standing balance neuroprosthetics for individuals with iSCI.

### Limitations

A potential limitation in the comparison of postural sway between TAon and TAoff periods was the assumption that COPv and COMa are rather stationary signals, and consequently their calculated variabilities should be less depending on their data lengths. However, this was not the case, as the COPv and COMa would have been lower during the TA*on* periods if the data segment lengths had influenced these results (Fig. 5).

Lastly, it was necessary for the safety of our participants with iSCI to wear shoes while performing the standing trials. Consequently, our AB-group wore shoes as well to create similar scenarios. Although some studies utilizing high heels suggest type of shoe affects the torque and COP^63,64^ our participants were instructed to wear comfortable walking shoes whose sole height and stiffness did not vary much. Thus, our participants all wore similar styled/type shoes which may affect our between participant differences, but we believe that this should not impact our conclusion on the effect of co-contractions on postural sway as these were within participant comparisons.

## Conclusions

The current study demonstrated that individuals with iSCI utilize co-contractions of the plantarflexor and dorsiflexor muscles more often than AB individuals, which corresponds with an increase in postural sway. These co-contractions may be a compensation strategy for individuals with iSCI to accommodate for decreased motor function, but the large increase of TA activity during co-contraction may result in increased ankle mechanical joint stiffness and consequently postural sway.

## Supplementary Information

Supplementary Information.

## Data Availability

The datasets generated during and/or analyzed during the current study are available from the corresponding author on request.
