# Publisher Correction: Co-contraction of ankle muscle activity during quiet standing in individuals with incomplete spinal cord injury is associated with postural instability

**DOI:** 10.1038/s41598-021-02784-0

**Published:** 2021-11-30

**Authors:** Kai Lon Fok, Jae W. Lee, Janelle Unger, Katherine Chan, Kristin E. Musselman, Kei Masani

**Affiliations:** 1 Institute of Biomedical Engineering, University of Toronto, Toronto, ON Canada; 2 KITE-Toronto Rehabilitation Institute, University Health Network, Toronto, ON Canada; 3 Rehabilitation Sciences Institute, University of Toronto, Toronto, ON Canada; 4 Department of Physical Therapy, University of Toronto, Toronto, ON Canada

Correction to: *Scientific Reports* https://doi.org/10.1038/s41598-021-99151-w, published online 01 October 2021.

The original version of this Article contained errors in Figure 6, panel (a). Under the EO and EC conditions, “Sim-AB” and “Sim-iSCI” were incorrectly given as “Si AB” and “Si SCI”. The original Figure 6 and accompanying legend appear below.

**Figure 6:**
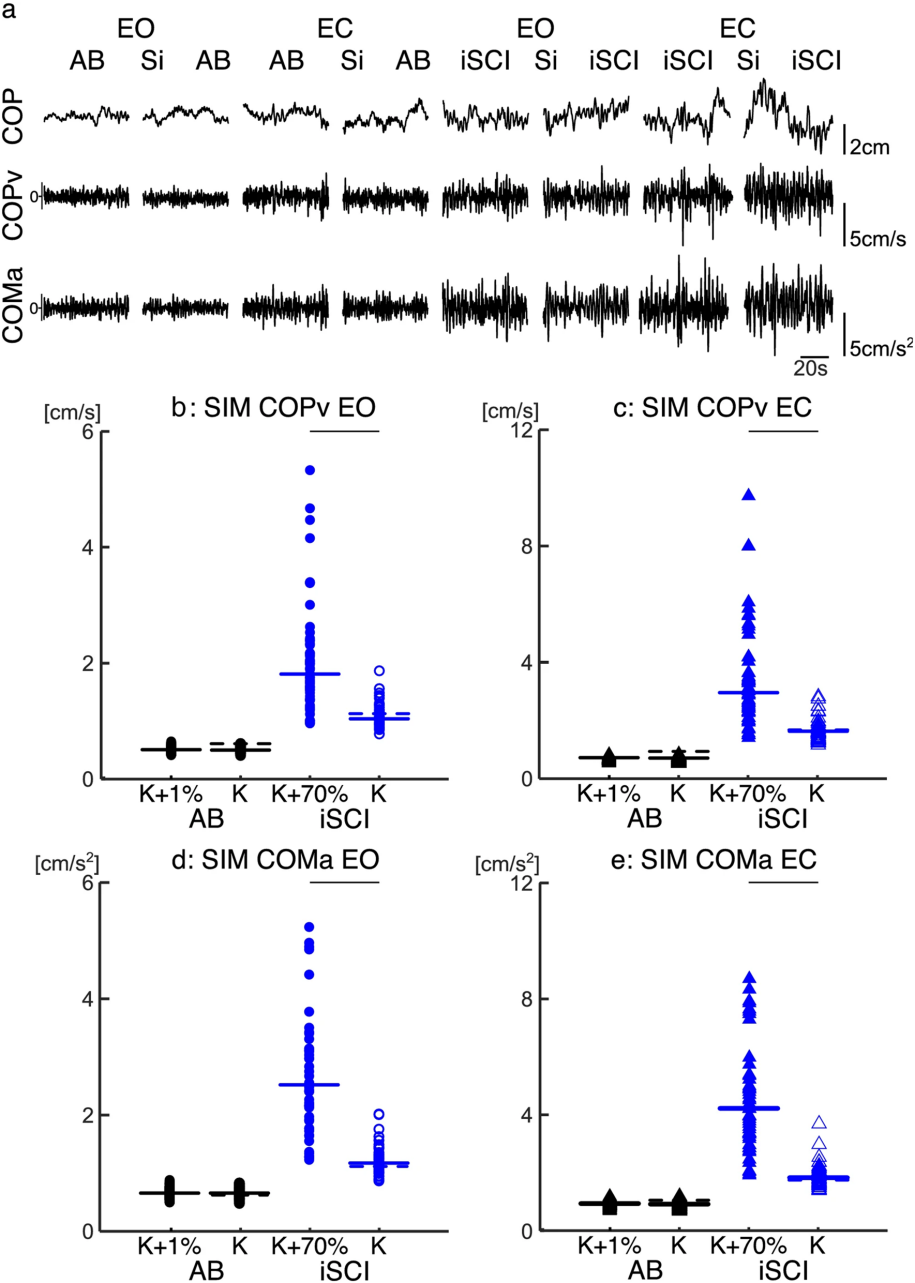
Example of the simulated postural sway (standard deviation of the COPv and COMa), and the comparison of an experimentally matched postural sway to postural sway due to an increased mechanical stiffness. (**a**) Representative 60 s traces for the experimental (AB/iSCI) and simulated (Sim-AB/Sim-iSCI) COP, COPv and COMa in both EO and EC conditions. (**b**) Distribution of the fluctuation of the COPv in the EO condition. (**b**) Distribution of the fluctuation of the COPv in the EC condition. (**c**) Distribution of the fluctuation of the COMa in the EO condition. (**d**) Distribution of the COMa in the EC condition. Simulation data presented for the Sim-AB (black, N = 137) and Sim-iSCI (blue, N = 55) groups in both EO (circles) and EC (triangles) conditions for periods of co-contraction (K + %—filled shapes) and no–co-contraction (K—empty shapes). Thick horizontal lines represent the group median, horizontal dashed lines represent the target experimental postural sway data used to determine the gains sensory and internal noise for the simulations. Individual data are plotted. Thin horizontal lines above the plots represent significant differences (p < 0.05).

The original Article has been corrected.

