# Supplementary material for: Co-contraction of ankle muscle activity during quiet standing in individuals with incomplete spinal cord injury is associated with postural instability
Source: Sci Rep. 2021 Oct 1;11:19599. doi: 10.1038/s41598-021-99151-w (PMC8486862; doi:10.1038/s41598-021-99151-w)
Supplement: Supplementary file 1 — Supplementary Information. [file 41598_2021_99151_MOESM1_ESM.pdf]

Supplemental information

Co-contraction of ankle muscle activity during quiet standing in individuals with incomplete spinal cord injury is associated with postural instability

Kai Lon Fok, Jae W. Lee, Janelle Unger, Katherine Chan, Kristin E. Musselman, Kei Masani

Supplemental Methods

Inclusion criteria

Individuals were included in the study based on the following criteria: ≥18 years of age; traumatic or non-progressive non-traumatic cause of injury; injury or onset of neurological symptoms occurred >1 year prior; injury rated C or D on the American Spinal Injury Association Impairment Scale (AIS); moderate level of trunk control (i.e. ability to reach forward >5 cm with an outstretched arm in standing); and no condition, other than their SCI, that affected walking or balance ability (e.g. stroke). Individuals were excluded from the study if they had severe contractures or spasticity in the lower extremities that interfered with maintaining an upright posture in standing.

MVC

Two 10-second trials were collected for each muscle group and allowed for 5 min of rest between the two trials. For each trial, participants were instructed to ramp up the contraction, hold the maximal contraction for 3-4 seconds and ramp back down. The contractions were resisted by the research team members. All contractions were performed in a seated position. The placement of resistance and orientation of the lower limbs during the MVC trials are summarized in Supplementary Table S1. A summary of the measured MVC is presented in Supplementary Table S2.

Data Analyses

The ratio of the muscle activity level during TA*on* to TA*off* of the MG, SOL and TA (%MVC on/off ratio) were calculated for each group and condition as a measure of how much each muscle was active during periods of TA*on*. The %MVC on/off ratios were non-normally distributed. Thus, for the %MVC on/off ratio, a Wilcoxon's rank-sum test (non-paired) was used to compared significant differences between groups, for each condition respectively.

Simulation Study

The different K increases were due to the observed differences in the experimental data where the iSCI-group's TA %MVC on/off ratio was about 1.68 times larger than in the AB-group.

Supplemental Results

Muscle activity

Supplementary Figure S2 shows the period of the MG and SOL activity as well as of co-contraction of the MG and TA. Overall, both AB- and iSCI-groups demonstrated similar periods of MG and SOL activity than the AB-group. Specifically, the %On was not significantly different between the AB- and iSCI-groups for the MG and SOL for each condition (MG: EO – $p = 1.00$, EC – $p = 1.00$;

SOL: EO – p = 1.00, EC – p = 0.673) (Supplementary Fig. S2A and B). Further, the %CC was significantly larger in both iSCI-group than in the AB-group (MG/TA: EO – p = 0.004, EC – p = 0.016 in both EO and EC conditions (Supplementary Fig. S2C).

Supplementary Figure S3 shows the ratio of the %MVC during periods of TAon and TAoff for each muscle. Overall, the MG and SOL %MVC on/off ratio was similar during periods of TAon and TAoff in both AB- and iSCI-groups, the TA %MVC on/off ratio was larger in the iSCI-group compared to the AB-group in the EC (p = 0.034) but not EO condition (p = 0.065). Also, the MG and SOL %MVC on/off ratio was not significantly different between the AB- and the iSCI-groups in each condition (MG: EO – p = 0.449, EC – p = 1.00; SOL: EO – p = 0.543, EC – p = 1.00) (Supplementary Fig. S3A and B).

Both the AB-group and the iSCI-group's MG and SOL %MVC on/off ratios were not significantly different from 1 (MG: AB EO – p = 0.256, AB EC – p = 0.277, iSCI EO – p = 1.00, iSCI EC – p = 0.883; SOL: AB EO – p = 0.473, AB EC – p = 0.628, iSCI EO – p = 0.525, iSCI EC – p = 0.883) (Supplementary Fig. S3A and B). For the AB-group, the TA %MVC on/off ratio was significantly different from 1 in the EO (p = 0.036) and EC (p = 0.015) conditions. For the iSCI-group, the TA %MVC on/off ratio was significantly greater than 1 in both EO (p = 0.023) and EC (p = 0.015) conditions (Supplementary Fig. S3C).

Effect of TA activation on postural sway

Supplementary Figure S4 shows the results of single leg COPv and COMa during TA*on* and TA*off*. During TA*on*, the AB-group demonstrated no changes in postural sway fluctuations; however, the iSCI-group's postural sway increased significantly. Specifically, the COPv was not significantly different between TA*on* and TA*off* in the AB-group in EO (p = 1.00) and EC (p = 1.00) conditions. In the iSCI-group, during TA*on* the COPv was significantly larger than during TA*off* in EO (p = 0.023) but not EC (p = 0.101) conditions (Supplementary Figure S4A and B). The COMa was not significantly different between TA*on* and TA*off* periods for the AB-group in EO (p = 1.00) and EC (p = 1.00) conditions. Further, in the iSCI-group, during TA*on* periods, the COMa was significantly larger than during TA*off* periods in EO (p = 0.023) but not EC (p = 0.057) conditions.

Supplemental Tables

Table S1 – Summary of posture and resistance used for MVC collection

Muscle	Position	Resistance	Action
Tibialis Anterior	Sitting	Dorsum of foot	Dorsiflexion
Medial Gastroc	Sitting; knee ext.	Ball of foot	Plantar flexion
Soleus	Sitting; knee flex.	Ball of foot	Plantar flexion

Table S2 – Summary of absolute MVC values (mV) for the MG, SOL and TA, for both AB- and iSCI-groups.

	AB			iSCI		
Participant	MG	SOL	TA	MG	SOL	TA
1	0.384	0.456	1.267	0.549	0.479	1.120
2	0.900	0.919	1.526	0.547	1.392	0.923
3	0.498	0.506	1.642	0.509	0.580	1.057
4	0.448	1.368	1.353	0.124	0.241	0.793
5	0.587	0.641	0.940	0.310	0.587	0.180
6	0.351	0.637	0.816	0.562	0.355	0.891
7	0.609	0.637	0.760	0.202	0.464	2.494
8	0.461	0.716	0.940	0.202	0.208	0.399
9	0.315	0.717	1.002	0.455	0.223	0.442
10	0.527	0.699	1.375	1.169	1.312	2.263
11	0.345	0.837	0.880	0.135	0.178	0.605
12	0.282	0.727	0.903	0.368	0.669	1.386
13	0.518	0.489	1.171	0.088	0.304	0.719

Table S3 - Summary of the median duration of TAon during quiet standing for the AB-, and iSCI-group. Values are reported in seconds, followed by the range. + represents that there was either only one instance of TAon or no instances of TAon.

	EO		EC	
	**AB**	**iSCI**	**AB**	**iSCI**
Participant	Median (range)	Median (range)	Median (range)	Median (range)
1	0.250+	0.830 (0.745 – 1.330)	0.970+	0.753 (0.050 – 6.535)
2	0+	0.345 (0.060 – 0.925)	0+	2.490 (0.080 – 26.010)
3	0+	1.210 (0.230 – 20.045)	0+	0.600 (0.025 – 1.550)
4	0+	1.208 (0.155 – 4.260)	0+	0.805 (0.020 – 4.620)
5	0.390 (0.070 – 1.520)	60.000+	0.950 (0.145 – 7.156)	60.000+
6	0+	0.813 (0.100 – 10.645)	0.105+	4.815 (0.465 – 15.215)
7	0.795 (0.290 -1.300)	1.650 (0.380 – 17.230)	1.015 (0.675 -60.000)	1.835 (0.380 – 13.895)
8	2.055 (0.470 -54.76	59.973 (59.945 - 60.000)	1.005 (0.430 -53.700)	59.973 (59.945 – 60.000)
9	0.505 (0.120 -3.305)	60.000+	0.373 (0.065 -0.755)	60.000+
10	0+	19.205 (0.390 - 37.190)	0+	33.290 (26.585 – 60.000)
11	0.380 (0.060 -0.740)	29.863 (14.745 – 44.950)	0.533 (0.070 -1.840)	3.280 (0.620 – 13.040)
12	0+	0+	1.563 (0.270 -11.125)	0.738 (0.475 – 0.845)
13	1.160 (0.005 -1.675)	5.718 (1.345 – 31.345)	1.910 (0.345 -3.895)	2.305 (0.155 – 18.520)

Supplemental Figures

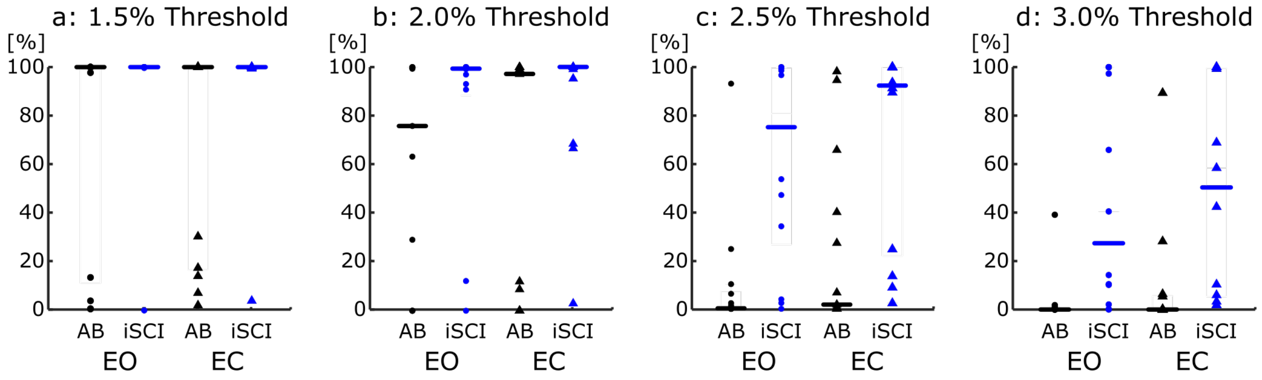

Figure S1 – Distribution of the percentage of the quiet standing trial where the TA activity is greater than certain TA %MVC thresholds for the AB-group (black) and the iSCI-group (blue). (A) 1.5%MVC (B) 2.0%MVC (C) 2.5%MVC (D) 3.0%MVC.

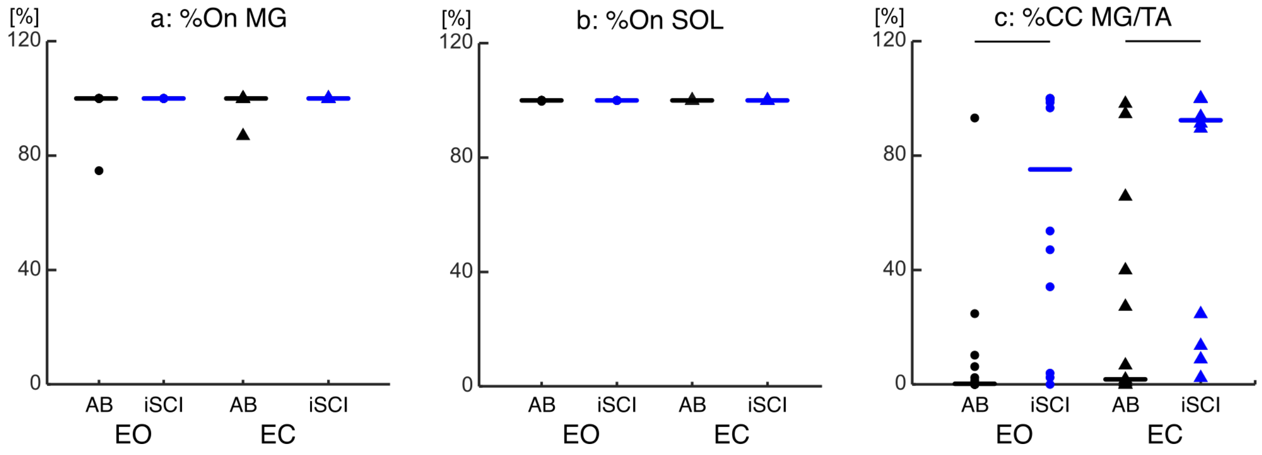

Figure S2 – Group data presented for the AB- (black) and the iSCI-group (blue) in both EO and EC conditions. Individual participant data are plotted as circles for the EO condition and triangles for the EC condition. N = 13 for AB-group, and N = 12 for iSCI-groups. Thick black, blue, and red horizontal lines represent the group median. Thin black horizontal bar indicates a p-value < 0.05. (A) Distribution of the mean amount of time MG was active as a percentage. (B) Distribution of the mean amount of time SOL was active as a percentage. (C) Distribution of the mean amount of time the MG and TA were active as a percentage.

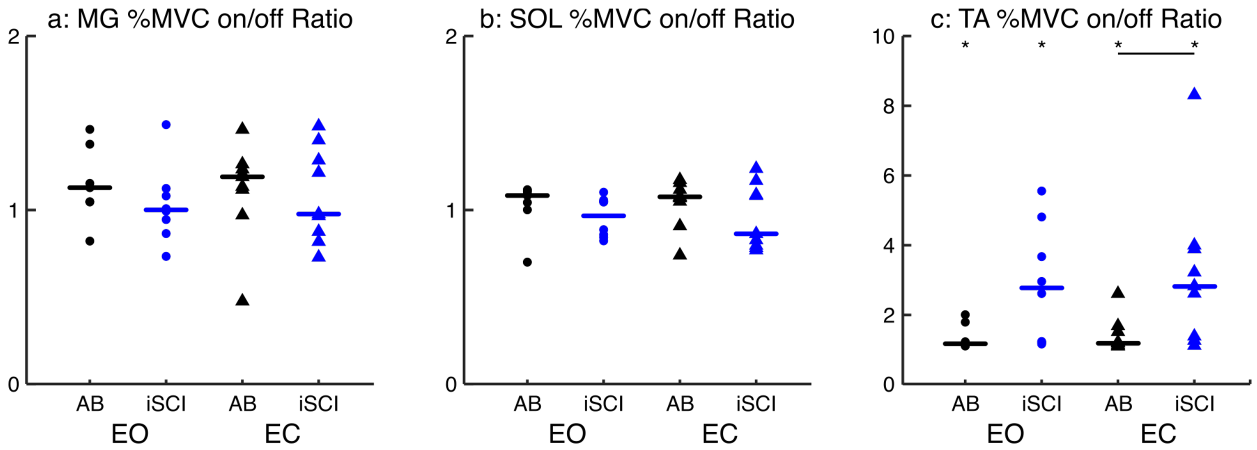

Figure S3 - Group data presented for the AB-group (black) and the iSCI-group (blue) in both EO (circles) and EC (triangles) conditions. Thick horizontal lines represent the group median. Individual participant data are plotted. Thin lines connecting points represent the same participant. The horizontal line above the plot indicates a significant difference between the groups and * denote a significant difference from 1 ($p < 0.05$). (A) Distribution of the mean ratio of the MG %MVC during cocontraction vs no cocontraction. (B) Distribution of the mean ratio of the SOL %MVC during cocontraction vs no cocontraction. (C) Distribution of the mean ratio of the TA %MVC during cocontraction vs no cocontraction.

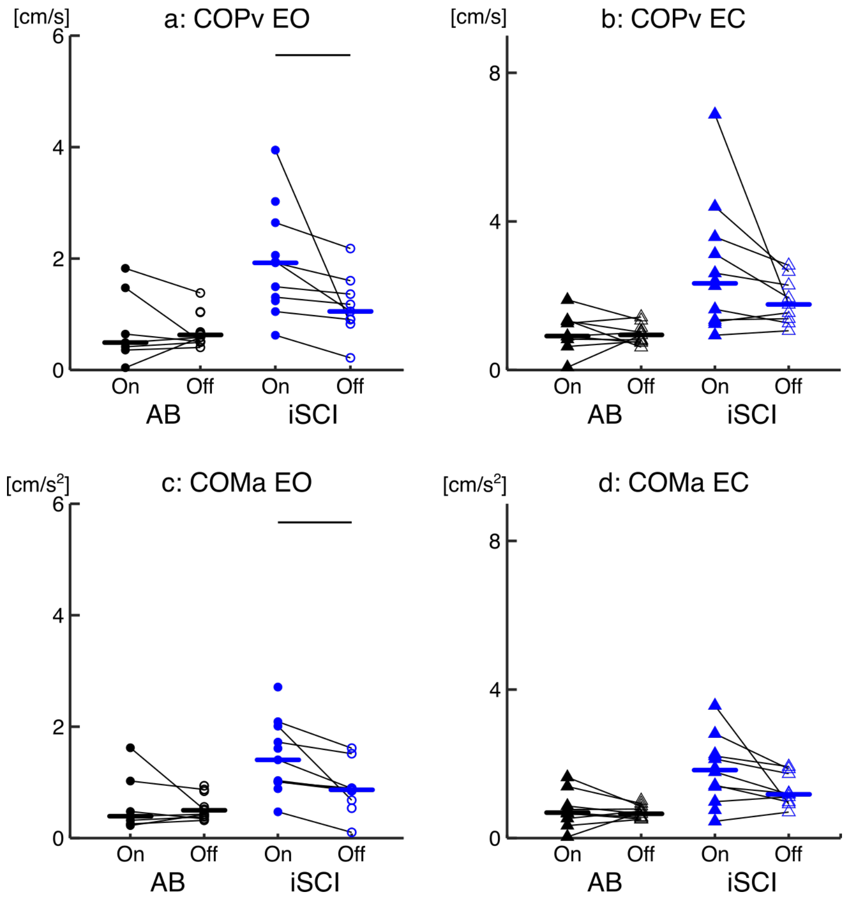

Figure S4 - Group data for the COPv and COMa for a single leg presented for the AB-group (black) and the iSCI-group (blue) in both EO (circles) and EC (triangles) conditions for periods of cocontraction (On – filled shape) and no-cocontraction (Off– open shape). Horizontal lines represent the group median. Each point represents an individual participant's data, thin black lines between data points connect the same participant and thin horizontal lines above the plots represent significant differences ($p < 0.05$). Thick horizontal lines within data points represent group medians. (A) Distribution of the fluctuation of the COPv in the EO. (B) Distribution of the fluctuation of the COPv in the EC. (C) Distribution of the fluctuation of the COMa in the EO. (D) Distribution of the COMa in the EC condition.
